# Perspective determines the production and interpretation of pointing gestures

**DOI:** 10.3758/s13423-020-01823-7

**Published:** 2020-10-15

**Authors:** Oliver Herbort, Lisa-Marie Krause, Wilfried Kunde

**Affiliations:** Department of Psychology, Julius-Maximilians-Universität Würzburg, Röntgenring 11, Würzburg, Germany

**Keywords:** Pointing gestures, Pointing production and interpretation, Deictic reference, Virtual reality

## Abstract

Pointing is a ubiquitous means of communication. Nevertheless, observers systematically misinterpret the location indicated by pointers. We examined whether these misunderstandings result from the typically different viewpoints of pointers and observers. Participants either pointed themselves or interpreted points while assuming the pointer’s or a typical observer perspective in a virtual reality environment. The perspective had a strong effect on the relationship between pointing gestures and referents, whereas the task had only a minor influence. This suggests that misunderstandings between pointers and observers primarily result from their typically different viewpoints.

## Introduction

Pointing gestures are ubiquitous in human communication and play an increasing role in human-robot interactions or interactions in virtual environments (Butterworth, 2003; Roth 2001; Wong & Gutwin, 2014). While pointing gestures are complemented by speech in some situations, people tend to rely mostly on pointing in other situations, for example when referring to non-salient objects at hard-to-describe positions – such as a star in the night sky or an animal hidden in the landscape. However, in such situations it also becomes apparent that initial attempts to rely mainly on pointing often fail to guide another person’s attention to a referent.

**Fig. 1:**
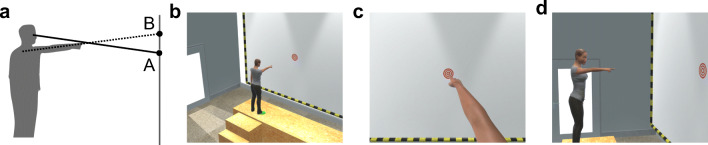
(**a**) A pointer who intends to indicate position A is typically believed to point at position B when watched from the side. (**b-d**) The screenshots show an overview of the virtual reality environment (**b**), the pointer perspective (**c**), and the observer perspective (**d**). The red-and-white disk either served as a referent for pointing or was used by participants to mark the pointed-at position

One reason why it is hard to communicate a precise location with pointing gestures is that pointing gestures are systematically misinterpreted by observers (Bangerter & Oppenheimer, 2006; Herbort & Kunde, 2016; Wnuczko & Kennedy, 2011). For example, observers typically judge pointing gestures to be directed at a higher position than intended by the pointer, apparently because the production and interpretation of pointing gestures accord to different geometric rules. An example is shown in Fig. 1a: Pointers typically put the index finger on the line between their eyes and the referent. In contrast, observers tend to extrapolate the line defined by the arm and fingers when seeing a pointer from the side, thus overestimating the referent’s height.

This raises the question why the production and interpretation of pointing gestures follow different rules. Many factors could potentially elicit differences in pointing production and interpretation. For example, pointers and observers differ with respect to their knowledge of the referent’s location, might attend different locations, and produce different types of movements. In this paper, however, we specifically test the hypothesis that differences in the production and interpretation of pointing gestures mainly result from the necessarily different perspectives of pointers and observers.

This assumption is suggested by a number of previous observations. First, pointing movements made while looking at one’s image in a mirror, or with eyes closed, differ from natural pointing movements (Herbort & Kunde, 2016; Wnuczko & Kennedy, 2011). One possible interpretation of these findings is that pointing production depends on the precise perception of the own pointing gesture and its relation to the referent and that this perceived relationship depends on the pointer’s perspective. Second, changes in the observer’s perspective affect the interpretation of pointing gestures. In one experiment, pointing gestures seen from the right were interpreted differently than gestures seen from the left (Bangerter & Oppenheimer, 2006). However, this apparent effect of the perspective has to be interpreted with care, because pointers always pointed with the hand close to the observer and their points with the left and right arm might have differed (cf. Cooney, Brady, & McKinney, 2018). Moreover, whereas observers typically interpret points as indicating a higher position than intended by the pointer (Bangerter & Oppenheimer, 2006; Herbort & Kunde, 2016, 2018; Wnuczko & Kennedy, 2011), such errors were rare in an experiment in which participants interpreted videos of pointing gestures recorded from the pointer’s viewpoint (Akkil & Isokoski, 2016). In sum, these findings suggest that both pointing production and interpretation depend on the perspective. Therefore, the necessarily different perspectives of pointers and observers might be a reason for pointer-observer misunderstandings.

To systematically address this question, we orthogonally manipulated the participant’s task and the perspective taken. That is, participants took the role of pointer or observer and thereby looked through the pointer’s or the observers’ eyes in a virtual reality (VR) setup. We expect that the way participants relate pointing gestures to referent positions is strongly affected by the perspective, whereas the task has no or only a minor effect on this relationship.

In this paper, we primarily address whether perspective and task affect the gesture-referent relationship and not how this relationship can be best described. Nevertheless, the findings that pointers typically align eye, index-finger, and referent and that observers on the side typically extrapolate the vector defined by the pointing arm and finger (Herbort & Kunde, 2016; Wnuczko & Kennedy, 2011) allow for more specific predictions. That is, eyes, index finger, and referent form a line when participants assume the pointer perspective, regardless of whether they point or interpret (Fig. 1a, solid line). Conversely, when pointers or observers see the pointer from the side, shoulder, index finger, and referent should fall on a line (Fig. 1a, dotted line). Although this model is certainly an oversimplification (Herbort & Kunde, 2016), it might be a helpful heuristic for the interpretation of our data. Hence, the gesture-referent relationships derived from these predictions will be shown alongside the empirical data and considered in the discussion, but we do not aim to formally test or elaborate this model.

## Method

### Participants

Twenty-four women age 18–40 years (mean age 23 years; 23 right-handed, one ambidextrous) completed the experiment after signing informed consent and were compensated with course credit or money. They were on average 168 cm tall (*SD* = 7 cm). The data were collected as part of a practical course at the Julius-Maximilians-Universität Würzburg, which limited the time available for data collection. To enable counterbalancing of the order of conditions and meet the time constraints, the sample size was set to 24 participants. As our virtual pointer was female, only data of female participants was collected. Considering the large effect sizes of systematic errors (e.g., vertical misunderstandings of naïve pointers and observers when pointing at referents 2 m away from the pointer yielded effect sizes of *d*_*z*_ = 2.8 and 3.3 in the experiments reported by Herbort & Kunde, 2018), a sample size of 5 would suffice to find the hypothesized effect of the perspective on pointing production and interpretation with a power of 1-β = .994. The experiment was conducted in accordance with the standards of the ethics commission of the Department of Psychology of the Julius-Maximilians-Universität Würzburg.

### Stimulus, apparatus, and procedure

Participants wore an HTC Vive head-mounted display (HMD) that immersed them in a virtual room (Fig. 1b). In the room, a virtual pointer stood on a podium (1 m high), facing a 5 m × 5 m white screen at a distance of 2 m. The position of the virtual viewpoint was fixed regardless of the actual movements of the participant but the orientation of the virtual viewpoint was aligned with participants’ actual head rotations. That is, participants could look in all possible directions in VR but always from one of two fixed positions. The virtual pointer always pointed with a stretched arm and with an extended index finger. The virtual pointer was 175 cm tall and thus slightly taller than the average participant.

In the pointing task, the referent at which participants were asked to point was a red and white disk (diameter 25 cm) on the white screen (Fig. 1 b–d). They held an HTC Vive controller in the right hand, which translated their real arm movements into movements of the virtual pointer’s right arm. This was realized by setting the direction of the virtual pointer’s arm to the direction of the vector from the virtual pointer’s shoulder to the hand-held controller. Although this did not result in a perfect mapping between the participants’ and virtual pointers’ postures, pointing felt natural and no participant reported problems controlling the arm. Participants pulled the controller’s trigger button once they pointed at the referent. After a 500-ms interval in which the screen faded to gray, the next trial was initiated.

In the interpretation task, the virtual pointer was presented in one predefined, static posture throughout each trial. Participants then moved the red and white referent disk to the location they believed the pointer was referring to (referent position) with the directional controls of an Xbox gamepad. Then, they pressed a gamepad button with the right index finger. Once the responses were given, the view turned gray for 500 ms and the next trial was started.

When participants assumed the pointer perspective, the participant viewed the scene through the eyes of the virtual pointer (Fig. 1c). The observer perspective placed the viewpoint to a position 3.5 m to the pointer’s right (Fig. 1d). The heights of both viewpoints were identical.

The experiment was split into four blocks, in each of which a specific combination of perspective and task was administered. Block order was counterbalanced over participants. In interpretation blocks, the pointer’s arm was presented once with each possible combination of the azimuths -27°, -21°, -15°, - 9°, -3°, 3°, 9°, 15°, 21°, and 27° and the elevations -15°, -10°, -5°, 0°, 5°, 10°, 15°, 20°, 25°, and 30°. An azimuth and elevation of 0° corresponded to an arm orientation that was perpendicular to the white screen. In pointing blocks, the referent was displayed once at each combination of the x-coordinates -150 cm, -117 cm, -83 cm, -50 cm, -17 cm, 17 cm, 50 cm, 83 cm, 117 cm, and 150 cm and the y-coordinates -120 cm, -93 cm, -67 cm, -40 cm, -13 cm, 13 cm, 40 cm, 67 cm, 93 cm, and 120 cm, where zero refers to a position centrally in front of the pointer and 270 cm above the floor (roughly at pointer’s eye height). Positive values denote right or upward arm orientations or positions. Trials were presented in pseudorandom order. In total, 400 trials were presented. Participants spent approximately 40 min in VR.

### Data analysis

All trials were entered in the analysis. To compare the relationship between gestures and referent positions across tasks, they were parameterized by linear regressions of the arm azimuth on the referent x-position and of the elevation on the referent y-position. Regressions were computed for each participant, task, and perspective. The regression allowed us to compare the gesture-referent relationships between tasks because they were “blind” as to whether the arm orientation was produced or interpreted and whether the referent was the to-be-pointed at position or the interpretation of a pointing gesture. The regressions were then analyzed in two ways. First, to identify differences between conditions, the slopes and intercepts of the regressions were compared with repeated-measure ANOVAs. Second, to directly test whether the task or the perspective had the greater influence, we compared actual pointing postures with predictions derived from conditions with the same task but the other perspective or vice versa.

## Results

### Description of effects

**Fig. 2:**
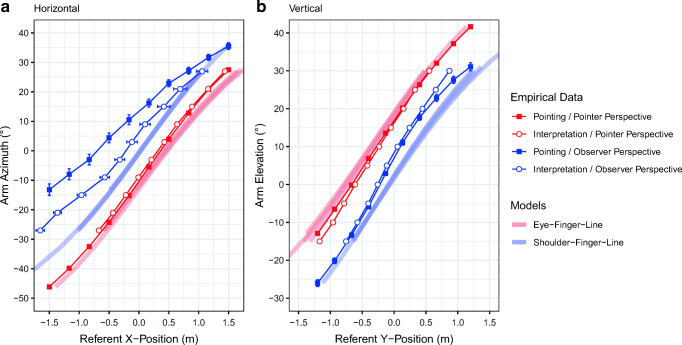
Figures plotting the mean arm azimuths against the mean referent x-positions (**a**) and the mean arm elevations against the mean referent y-positions (**b**) for each condition. Positive values indicate rightward and upward arm orientations or positions. Error bars show ± 1 SEM and are sometimes shrouded by the markers. The model predictions were derived by averaging the trial-wise extrapolation of the eye-finger vector and shoulder-finger vector. The eye position was defined as the head-mounted display position in the pointer-perspective conditions and as the point between the virtual pointer’s eyes in the observer-perspective conditions

Figure 2a and b show the relationships between gestures and referent positions for all experimental conditions. The bold lines in the charts show geometric eye-finger extrapolation and shoulder-finger-extrapolation for comparison. We first describe the outcomes and then report the statistical analyses. Not surprisingly, arm postures and the referent positions were closely related in each condition and dimension. Note that we first describe the vertical dimension (Fig. 2b) because of the simpler data pattern and then inspect the horizontal dimension. The relationships between arm elevations and referent y-positions depended strongly on the perspective but were not substantially affected by the task. More specifically, more upward-pointing gestures were produced from the pointer perspective than from the observer perspective. Conversely, interpretations from the observer perspective resulted in higher referent positions than the interpretation of the same gestures from the pointer perspective. Pointing and interpretations from the pointer perspective were very well captured by the eye-finger-extrapolation model. From the observer perspective, pointing and interpretation were reasonably well described as extrapolation of the shoulder-finger line. Thus, the data from the vertical dimension support our initial hypothesis that the typically different perspectives of pointers and observers are the primary cause of misunderstandings.

The pattern is less clear for the horizontal dimension (Fig. 2a). When assuming the pointer perspective, the curves from the pointing and interpretation condition overlapped and were closely approximated by the eye-finger model. Moreover, they differed clearly from both curves of the observer-perspective conditions, in which arm orientations were more rightward. When participants took the observer perspective, the curve of the pointing condition differed from that of the interpretation condition. That is, arm azimuths were biased more rightward during pointing than during interpretation. While the data are better approximated by shoulder-finger extrapolation than eye-finger extrapolation, arm azimuths are generally more rightward than predicted by the model.

### Comparison of regression parameters

**Fig. 3:**
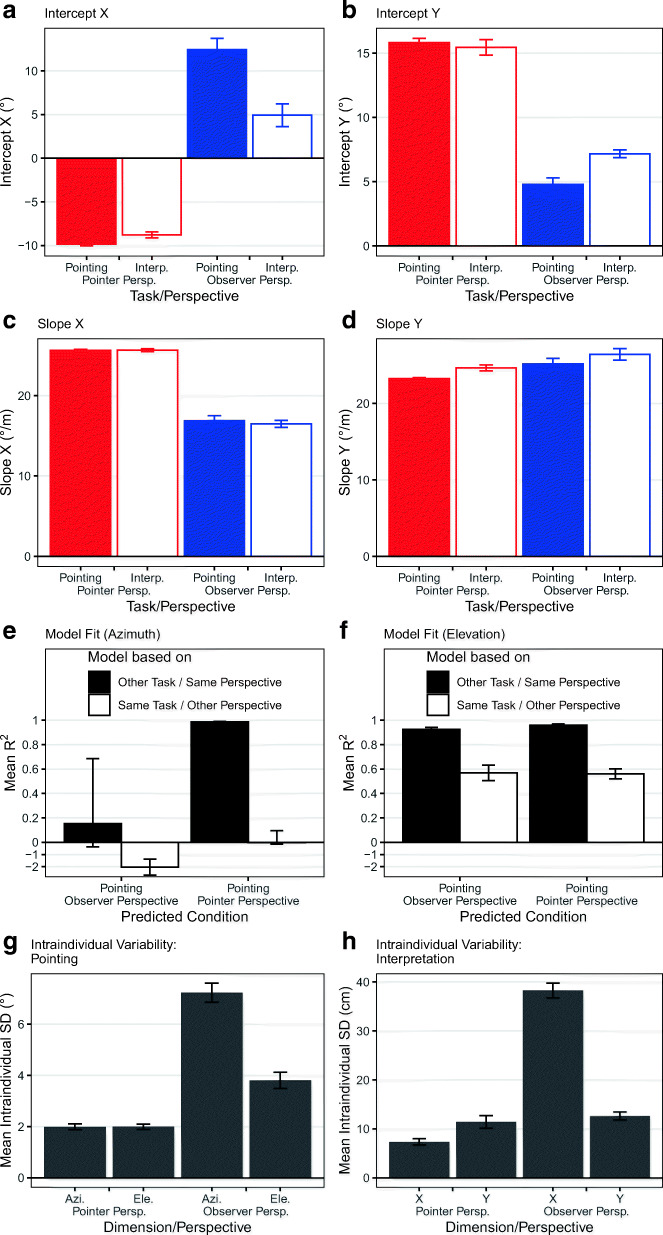
(**a–d**) Charts showing the intercepts (**a, b**) and slopes (**c, d**) of the linear regressions for the horizontal and vertical dimension. The colors of the bars are matched to Fig. 2. (**e**–**f**) Charts showing how well pointing gestures can be predicted from the linear regression models derived from the same task but the other perspective or vice versa. Note that the y-axis is compressed for negative R^2^s. (**g**–**h**) The charts show the mean intraindividual variability in all conditions. Error bars show ± 1 SEM

To support the above description statistically, we compared the intercepts and slopes of the participant-wise linear regressions computed for the curves presented in Fig. 2. Figure 3 (a–d) shows the parameters of the regressions. The intercepts and slopes of the regressions were entered in repeated-measure ANOVAs with factors of task and of perspective. Table 1 provides the results. Significant results that survived the Bonferroni-Holm correction are marked with an asterisk.

**Table 1:** Results of ANOVA on regression parameters

	Task			Perspective			Interaction		
Parameter	*F*(1,23)	*p*	η^2^_p_	*F*(1,23)	*p*	η^2^_p_	*F*(1,23)	*p*	η^2^_p_
X intercept	17.8	< .001	.44*	268.2	<.001	.92*	28.0	<.001	.55*
X slope	0.2	.660	.01	686.3	<.001	.97*	0.3	.592	.01
Y intercept	6.4	.019	.22	479.0	<.001	.95*	15.1	.001	.40*
Y slope	7.1	.014	.24	10.4	.004	.31*	0.1	.790	.00

In the horizontal dimension, the factor perspective affected intercepts and slopes. The intercept was further affected by the task and the interaction. The interaction indicates that the task had a larger effect in the observer-perspective conditions than in the pointer-perspective conditions. The slope was not significantly affected by the task or an interaction between task and perspective.

In the vertical dimension, the factor perspective affected intercepts and slopes of the regressions. The interaction was significant for the intercept, because the task had a larger effect in the observer-perspective conditions than in the pointer-perspective conditions. There were no other significant effects.

In summary, the pointing perspective had a strong effect on how participants related pointing gestures to referents – thus confirming our hypothesis. However, when participants assumed the observer perspective, the task also had a relatively strong effect in the horizontal and a tiny effect in the vertical dimension.

### Relative effect of perspective and task

The previous section revealed an apparently strong effect of the perspective but sometimes also an effect of the task on the relationship between pointing gestures and referent locations. Next, we aimed to test which factor had the larger impact by checking whether data of conditions with the same task but different perspectives or different tasks but the same perspectives were more similar. To this end, we predicted the elevations and azimuths of the self-produced pointing gestures separately for both perspectives. Predictions were based on the participant-wise linear regressions derived for each individual condition. The goodness of fit of the predictions was quantified with the *R*^*2*^ statistic. Figure 2 (e–f) shows that the mean *R*^*2*^s for azimuths and elevations were higher when the pointing gestures were predicted based on conditions that used the same perspective but the other task (i.e., interpretation) than vice versa. This was confirmed by paired t-tests, all t(23) ≥ 5.6, all *p*s < .001, all d_z_ ≥ 1.1. Thus, the referent-posture relationship was more similar between conditions with different tasks and identical perspective than between conditions with the same task but different perspectives.

### Intraindividual variability

The task only had a notable influence in the horizontal dimension in the observer-perspective conditions. As we suspected that this was related to the higher visual ambiguity in these conditions, we decided (post hoc) to analyze the intraindividual standard deviations as a proxy for ambiguity. The standard deviations of azimuths (elevations) were computed for each participant, each perspective, and each referent y-position (x-position). The standard deviations of referent x-positions (y-positions) were computed analogously for each participant, each perspective, and each arm elevation (azimuth). Figure 2g shows that the mean intraindividual standard deviations of the azimuth were higher in the observer-perspective condition than in the pointer-perspective condition and also higher than the ambiguity of the elevations in both perspectives, all *t*(23) ≥ 10.5, all *p*s < .001, all *d*_*z*_ *≥* 2.14. Likewise, Fig. 2h shows that the referent x-position was more variable in the observer-perspective condition than in the pointer-perspective condition, and more ambiguous than the referent y-positions in the pointer perspective or observer-perspective condition, all *t*(23) ≥ 16.4, all *p*s < .001, all *d*_*z*_ *≥* 3.35.

## Discussion

We studied whether the spatial mapping between pointing gestures and pointed-at referents differs between pointing production and pointing interpretation because of the usually different perspectives of pointers and observers. This hypothesis was generally borne out by the data. First, the perspective had a rather large effect on both pointing production and interpretation. Second, a participant’s pointing gestures could be better predicted from her interpretations from the same perspective than from her pointing movements when assuming another perspective. In the following, we discuss the relationships between gestures and referents, the effect of the viewpoint, the effect of the task, and relate the current results to previous experiments.

While we did not set out to test the hypothesis that pointing and its interpretation exactly adheres either to eye-finger extrapolation or to shoulder-finger extrapolation, our data suggest that these methods were a good approximation in most conditions. In the observer-viewpoint conditions, the empirical data were very close to those predicted by eye-finger extrapolation. Eye-finger extrapolation predicted slightly higher and more leftward pointing gestures than actually observed. Hence, participants might not precisely align the centers of eye, index finger and referent, but rather position the index finger slightly below and to the right of the referent. Furthermore, as our model does not take eye dominance into account, a lateral offset of the model could be expected. Shoulder-finger extrapolation provides a reasonable match for the vertical dimension in the observer-perspective conditions. The steeper curve of the empirical data most likely resulted from participants being biased toward the horizontal axes when attempting to extrapolate pointing gestures (Herbort & Kunde, 2016). Finally, the shoulder-finger method did not provide a good approximation for the horizontal data in the observer condition. One possible reason is that participants did not only extrapolate the vector defined by arm and finger but also took the index finger position in their visual field into account. As a result, pointing gestures observed from the right would be interpreted as more leftward than predicted by the shoulder-finger-extrapolation method. Thus, the shoulder-finger method, which has so far been successful in describing pointing interpretation in the vertical dimension when the pointer is seen from the side (Herbort & Kunde, 2016; Wnuczko & Kennedy, 2011), needs further elaboration.

An interesting question is why pointers and observers rely on different visual cues when assuming different perspectives. An inspection of the pointer and observer perspective (Fig. 1 c–d) indicates two possible reasons. First, the position of the index finger in the visual field may be considered a plausible, easy-to-interpret cue when adopting the pointer perspective but it is obviously invalid – definitively in our experiment and arguably in many natural situations – when adopting the observer perspective. Second, arm and finger form a salient directional cue that can be easily extrapolated when seen from the side, but much less so when seen through the pointer’s eyes. Hence, we suggest that participants always use the most salient and easy to process cue to point or interpret points. However, these cues depend on the perspective.

Beside the viewpoint, the task also affected the gesture-referent relationship. However, this effect was only substantial for the horizontal component and only when participants assumed the observer perspective. The higher visual ambiguity in this condition could have allowed for an influence of the task in at least two not mutually exclusive ways. First, when participants classify visual stimuli with manual actions, such as lever movements, responses to ambiguous stimuli are biased toward whichever response has the lower motor costs (Hagura, Haggard & Diedrichsen, 2017; Marcos, Cos, Girard & Verschure, 2015). Our pointers faced a likewise task, in which they responded with manual actions to an ambiguous visual stimulus – in this case the horizontal position of the referent disk. As leftward points with the stretched arm resulted in relatively uncomfortable postures and thus had higher motor costs (Kee & Karwowski, 2004), pointing movements may have been biased rightward. As such, the rightward bias may be a direct result of the requirement to use the right arm for pointing. Whereas this assumption accords with the rightward bias being largest for points to the left, it cannot explain why the rightward bias prevailed for forward points or points to the right and can thus only be a part of the explanation. If this factor played a role, it would imply that pointers trade-off pointing accuracy with biomechanical costs but that observers do not take such trade-offs into account – thus causing systematic misunderstandings.

Second, the differences in slopes could be the result of the “regression effect” that is typically observed in psychophysical studies (Poulten, 1979; Stevens & Greenbaum, 1966). The regression effect denotes that psychophysical judgments are often biased toward a central point when the relationship between two quantities is at least somewhat ambiguous. If the productions of pointing gestures are biased toward a central posture and interpretations are biased toward a central location, differences in slopes of the relationship between referents and pointing gestures emerge necessarily (Stevens & Greenbaum, 1966). Again, while this assumption readily explains the differences in slopes, it does not offer a straightforward explanation for the general rightward bias. This explanation suggests that pointing production and interpretation differs because the translation of a known referent location into a pointing gesture and the translation of a perceived pointing gesture into a location are both subject to central tendency biases. In sum, this suggests that differences between the geometric rules underlying pointing production and pointing interpretation may be partially caused by the task, but only when it is difficult to perceive the orientation of the arm or the exact location of the referent.

The results clearly show that the interpretation of the same pointing gesture depends considerably on the perspective. This finding allows reconciliation of inconsistencies between previous studies. For example, we successfully modelled pointing interpretation as (biased) extrapolation of the arm and finger (Herbort & Kunde, 2016) without considering the relative position of pointer and observer. By contrast, Bangerter and Oppenheimer (2006) reported that the relative position of pointer and observer affected pointer-observer misunderstandings. The present results suggest that this discrepancy can be attributed to the different observer perspectives used in both studies: The relative position of pointer and observer should not have had a major effect in the former study, as participants saw the pointer from the side. By contrast, observer and pointer sat side by side in the latter study. Thus, the observer’s view was relatively close to that of the pointer, suggesting that observers interpreted points at least partly based on the precise position of the pointer’s index finger in their visual field. As the relative position of the pointer’s finger in the observer’s field of view depended on the side on which the pointer was sitting, interpretations should also have been affected by the pointer’s position. In summary, the differences in viewpoints may explain the different outcomes of previous studies on pointing interpretation.

Although conducted in VR with individual participants, the present study can be related to typical misunderstandings between human pointers and observers in the real world. For example, our participants pointed at a height of 0 cm with the arm elevated by approximately 17° when adopting the pointer’s perspective. However, when they watched the pointer from the side, an arm elevation of 17° would be interpreted as implying a height of 34 cm (*SD* = 11 cm). That is, participants would misinterpret their own pointing gestures if they pointed using the pointer’s perspective and interpreted their points from the observer perspective. The magnitude of this vertical error corresponds to misunderstanding between naïve pointers and observers in a comparable real-life setting (Herbort & Kunde, 2018).

Finally, the present experiment has implications for practical applications. Various methods have been proposed to enhance pointing-based communication in collaborative virtual environments or robot pointing (Wong & Gutwin, 2014). These approaches involve modifying the pointing gestures of another person’s avatar to counteract pointer observer misunderstandings (Sousa et al., 2019) or to generate robot pointing gestures that are optimized for legibility (Holladay, Dragan, & Srinivasa, 2014). Albeit such approaches already facilitate pointing-based communication, they might be further enhanced by taking an observer’s viewpoint into account. Considering pointing in virtual environments, the present experiments also suggest that allowing an observer to adopt a pointer’s viewpoint could make pointing a much more effective means of communication.
